# Analytical performance and visual evaluation of fentanyl and xylazine test strips

**DOI:** 10.1186/s12954-026-01415-z

**Published:** 2026-02-20

**Authors:** Alan H. B. Wu, Chui Mei Ong, Melissa Alamillo, Steven Farias, Luana Barbosa

**Affiliations:** 1 Department of Laboratory Medicine, University of California, San Francisco, USA; 2 Materic|, 1300 Bayard St, Baltimore, MD USA; 3 Brand New Holdings, Sheridan, WY USA

**Keywords:** Xylazine adulterant, Fentanyl, Fentanyl analogues, Harm reduction

## Abstract

**Background:**

Testing street drugs for the presence of active adulterants such as fentanyl and xylazine can provide the user some confidence as to the safety of their drugs.

**Methods:**

We obtained 3 different commercially available xylazine and 3 different commercially available fentanyl test strips and evaluated them for analytical sensitivity using drug standards. The specificity of the fentanyl strips against fentanyl analogues was also assessed. Powdered fentanyl, xylazine, and fentanyl analogue standards were dissolved in water and serially diluted to bracket the manufacturer’s stated test strip sensitivity. Each dilution was tested in duplicate until a negative result was obtained. The ability to discern positive from negative results under different lighting conditions was also assessed for one of the strips (two lots of reagents).

**Results:**

All three xylazine test strips detected the drug at concentrations below the manufacturer’s stated limits, however one strip was substantially more sensitive (at 50 ng/mL) than the other two (at 250 ng/mL). One of the fentanyl strips had the best sensitivity (at 3.5 ng/mL), well below the stated sensitivity of 20 ng/mL. The other two were less sensitive (at 7 and 250 ng/mL). For specificity against fentanyl analogues, all of the strips tested positive for all of the analogues tested, but at different levels. The visual endpoints for all of the strips were acceptable under normal lighting conditions, but cannot be read when the ambient light is low.

**Conclusion:**

Based on these results, we selected one xylazine (Shanghai Accurature Diagnostics) and one fentanyl strip (W.P.H.M.) based on the best analytical sensitivity. The fentanyl strip chosen had varying degrees of specificity against the other manufacturers.

## Introduction

The opioid crisis continues to rage on in the United States and other countries, leading to an unacceptable number of overdose deaths per year. The culprit drug has evolved from natural opiates such as morphine, to semi-synthetic opioids such as heroin and oxycodone, to fentanyl, a synthetic opioid that is 50 times more potent than heroin and 100 times more than morphine, and is responsible for the most death of recent years. A recent complication to this crisis is the introduction of xylazine as a fentanyl adulterant. Xylazine was developed and approved as a sedative for veterinarian purposes [1]. Adulteration of opioids with xylazine began in Philadelphia and has spread throughout the U.S. While xylazine use is not associated with increased mortality from opioids, it presence has been associated with open skin ulcerations [2].

Much of the morbidity and mortality of opioid abuse has been through the intentional adulteration of the drug supply with fentanyl which has created undetectable risks for people using drugs. Therapeutic drugs such as sedatives that are available by counterfeit pharmaceuticals have been adulterated with fentanyl and xylazine by clandestine manufacturers. As a means to curb the dangers of this practice, testing street drugs by users prior to use can ensure safety of the product prior to its use. The use of laboratory quality instrumentation such as Raman Spectroscopy, ion mobility spectrometry, or mass spectrometry is the gold standard in terms of sensitivity and accuracy [3]. However, such testing services are not available by the users themselves. This has led to the release of immunoassay test strips which can provide a result within minutes without instrumentation. The purpose of this study was to examine the sensitivity and specificity of drug test strips and an assessment of the visual endpoints for providing harm reduction services.

## Methods

### Drug standards

For analytical sensitivity studies, fentanyl and xylazine drug standards in powdered form were obtained from Cayman Chemical Company (Ann Arbor MI). For analytical specificity studies, the following fentanyl analogues were also obtained in powdered from Caymen Chemical Company (m-fluorobutyrylfentanyl, acetylfentanyl, acrylfentanyl, furanylfentanyl, cyclopropylfentanyl, B-OH thiofentanyl, butyrylfentanyl and tetrhydrofuranylfentanyl. Drugs were weighed using an analytical balance (Mettler Inc.) and dissolved in methanol to produce stock solutions of 25, 50, 100, 250, 500, 1000, 2000, 5000, and 10,000 ng/mL for xylazine, and 1.75, 3.5, 7, 14, 28, 56, 112, and 225 ng/mL for fentanyl. All serial dilutions were performed in water.

### Drug test strips

Xylazine test strips were purchased from three manufacturers: Shanghai Accurature Diagnostics (Strip #1), WPHM (Strip #2) and Co-Innovation BioTech (Strip #3). Fentanyl test strips were purchased from 3 manufacturers W.H.P.M. (Strip #4), Tianjin Mingao (Strip #5), and China Med (Strip #6). Each of these strips were originally designed for detecting the presence of xylazine or fentanyl in urine samples. The strips were dipped into test solutions for 5–15 s and removed. Each solution was tested in duplicate. After 5 min, visual readings were conducted by two operators, and photographs were taken of all strips tested. The production of a line in both the control and test area was interpreted as a negative result, i.e., no target drug present in the sample. The production of a line in only the control area was interpreted as a positive result. Strips that produced no lines was interpreted as an invalid test, and the strip was discarded. As a negative control, distilled water containing no drugs was tested for each lot of strips. Due to supply limitations, testing was not conducted on all of the prepared samples.

We tested the drug strips under different lighting conditions including indoors with windows in full daylight (DL), indoors without windows with lights (IL), indoors without windows, without lights and door closed (IL Off), indoors without windows, without lights and door ajar (IL Indirect), and indoors without windows, lights off, door closed, using cell phone flashlight as a light source (flashlight). The results were 1: control stripe, 2: C and T strips, and U: unclear or uncertain reading. Each strip was tested in duplicate by three different operators, with at least two who were present to read each strip.

## Results

Of all of the test strips tested, we did not find any failures, i.e., absence of a line in the control region. Table 1 shows the results of the sensitivity studies for 3 manufacturers of xylazine strips. Test strip #1 provided the most sensitive signal, with positive results occurring at 50 ng/mL. This is significantly lower than the stated manufacturer’s stated sensitivity of 450 ng/mL. Test strip #2 and #3 each turned positive at 250 ng/mL, which was also below the package insert specification of 300 and 1000 ng/mL, respectively.

**Table 1:** Drug sensitivity

Drug name	Drug concentration	Result		
		Test strip 1^a^	Test strip 2	Test strip 3
Xylazine	Blank	N/N	N/N	N/N
	25 ng/mL	N/N	N/N	x
	50	P/P	N/N	x
	100	P/P	N/N	N/N
	250	P/P	P/P	P/P
	500	P/P	P/P	P/P
	1000	P/P	P/P	x
	2000	P/P	P/P	P/P
	5000	P/P	P/P	x
	10,000	P/P	P/P	P/P

		Test strip 4	Test strip 5	Test strip 6
Fentanyl	Blank	N/N	N/N	N/N
	1.75 ng/mL	N/N	N/N	x
	3.5	P/P	N/N	x
	7	P/P	P/P	N/N
	14	P/P	P/P	N/N
	28	P/P	P/P	N/N
	56	P/P	P/P	N/N
	112	P/P	P/P	P/P
	225	P/P	P/P	P/P

^a^Results of duplicate measurements taken. N = negative, P = positive

Strip #1: Shanghai Accurature Diagnostics; #2: W.P.H.M; #3: Co-Innovation; #4: W.P.H.M,; #5: Tianjin; #6: China Med. N = negative result. P = positive result. N/N, negative. x=not tested

Table 1 also shows the sensitivity results for the 3 fentanyl test strips evaluated.

**Fig. 1:**
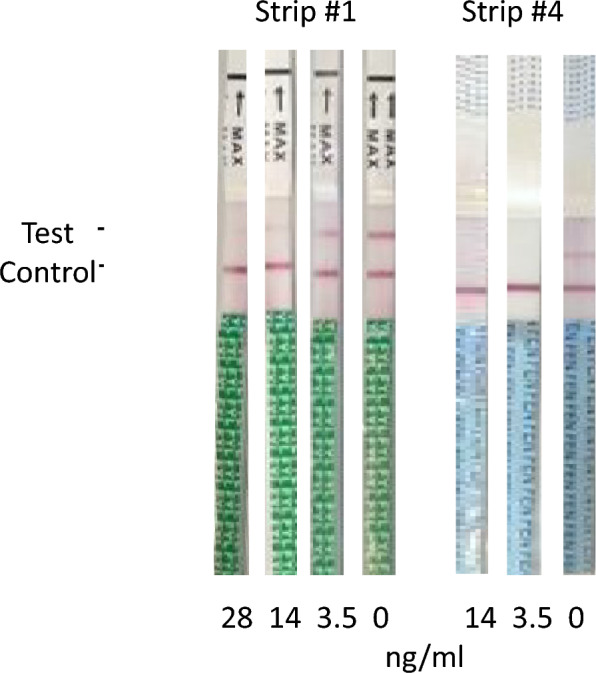
Head-to-head comparison of the Henso (left) and W.H.P.M (Lot #C3110310, right) test strips at different fentanyl durg concentrations. The Henso strip first turned positive at 29 ng/mL, compared to the W.H.PM. strip which turned positive at 3.5 ng/mL

Test strip #4 had the highest sensitivity, turning positive at 3.5 ng/mL (Fig. 1). These were all also considerably lower than what was stated in each of the package inserts of 20 ng/mL for all of the strips tested. Test strips #5 and #6 had lower sensitivity at 7 ng/mL and 112 ng/mL, respectively.

Table 2 shows the result of the specificity for the fentanyl strip against drug analogues. Due to limitations in the availability of test strips, not all drug analogues were tested on all devices. However, some generalizations can be made. The results show that all of the strips have some degree of cross-reactivity towards each of these fentanyl analogues. The results were not consistent as some strips had better specificity to some drugs but not others. Due to supply issues, we were not able to test the full range of fentanyl analogues for test strip #5, therefore no results are shown.

**Table 2:** Drug specificity

Drug name	Drug dilution	Results		
		Test strip 4	Test strip 5	Test strip 6
Meta-FluoroButylFentanyl	1:100 dilution	P/P	x	P/P
	1:1000 dilution	P/P	P/P	P/P
	1:10,000 dilution	N/N	N/N	P/P
	1:100,000 dilution	x	x	N/N
Acetyl fentanyl	1:100 dilution	x	x	P/P
	1:1000 dilution	x	P/P	P/P
	1:10,000 dilution	x	P/P	N/N
	1:100,000 dilution	x	N/N	x
Acryl fentanyl	1:100 dilution	P/P	x	P/P
	1:1000 dilution	P/P	P/P	P/P
	1:10,000 dilution	N/N	P/P	N/N
	1:100,000 dilution	x	N/N	x
Furanyl fentanyl	1:100 dilution	P/P	x	P/P
	1:1000 dilution	N/N	P/P	P/P
	1:10,000 dilution	N/N	P/P	N/N
	1:100,000 dilution	x	N/N	x
Cyclopropyl fentanyl	1:100 dilution	P/P	x	P/P
	1:1000 dilution	P/P	P/P	P/P
	1:10,000 dilution	N/N	P/P	P/P
	1:100,000 dilution		N/N	N/N
B-OH Thiofentanyl	1:100 dilution	P/P	x	P/P
	1:1000 dilution	P/P	P/P	N/N
	1:10,000 dilution	P/P	P/P	x
	1:100,000 dilution	N/N	N/N	x
Butyryl fentanyl	1:100 dilution	x	x	P/P
	1:1000 dilution	x	P/P	P/P
	1:10,000 dilution	x	P/P	N/N
	1:100,000 dilution	x	N/N	x
Tetrahydrofuranly fentanyl	1:100 dilution	x	x	P/P
	1:1000 dilution	x	P/P	P/P
	1:10,000 dilution	x	P/P	N/N
	1:100,000 dilution	x	N/N	x

Strip #4: W.P.H.M,; #5: Tianjin; #6: China Med. N = negative result. P = positive result. x=not tested

Table 3 shows the results of the visibility tests conducted. While each strip was tested in duplicate, there was no discrepancy between duplicate readings, therefore only one result is shown. The strips were readable and produced the same results under all of the study conditions except under the indoor without lights and the door is closed. Test strip #1 took longer to fully develop but was clear after 5 min.

**Table 3:** Results under different lighting conditions

Lighting conditions	Observer 1	Observer 2	Observer 3
WHPM (Lot #1)			
DL	2	2	2
IL	2	2	2
IL Off	U	U	U
IL Indirect	2	2	2
Flashlight	2	2	2
WHPM (Lot #2)			
DL	2	2	2
IL	2	2	2
IL Off	U	U	U
IL Indirect	2	2	2
Flashlight	2	2	2

2 = two lines. U = unreadable result

## Discussion

There have been several other studies on the analytical performance of drug test strips for fentanyl. Bergh et al. tested four commercial fentanyl strips and found sensitivities that were 5–10 times higher than what was stated in the package insert [4]. We observed similar results, although each of these strips were different from the ones tested in this study. For specificity, these investigators also found varying degrees of specificity against the fentanyl analogues tested. One unexpected finding was a positive result for ascorbic acid on one of the strips tested. Halifax tested only one commercial fentanyl test strip (BTNX, stated sensitivity, 20 ng/mL) but obtained five different lots [5]. For sensitivity, the reported variable results with some lots meeting the stated analytical sensitivity of 20 ng/mL while the sensitivity was 10 times lower (i.e., positive at 200 ng/mL). While they also found a variable degree of cross-reactivity towards fentanyl analogues, some lots were positive for other non-related drugs such as lidocaine, diphenhydramine, methamphetamine, and 3,4-methylenedioxymethamphetamine (Ecstasy).

There are fewer studies for xylazine as this adulterant has only recently emerged. Sisco et al. tested a xylazine test strip (BTNX, stated sensitivity 1000 ng/mL) and found mixed results at the cutoff [6]. This is unlike this report which found sensitivities greatly below this limit. The only false positive result was due to lidocaine at 100,000 ng/L. None of these non-structurally related drugs towards xylazine or fentanyl was testing in this study.

The availability of drug test strips can be effective in curbing the opioid crisis. In one study conducted in the Mid-Atlantic region of the U.S., the available of test strips led to a 23–69% of participants reported risk reduction behaviors such as using less drug than intended and going slower when using [7]. In another study, participants reported the prudent practice of having an opioid antagonist such as naloxone readily available [8]. Given the rapid onset of toxic effects, these can be life-saving measures.

Test strips are now widely available, inexpensive, and relatively easy to use. Reading the endpoints, however, requires some training. Unlike at-home pregnancy tests or COVID-19 antigen tests, the interpretation of results are reversed, i.e., the absence of a line in the test zone indicates a positive result. Therefore without proper instruction, there can be ambiguity of interpreting a faint line in the test zone as a positive result. Also, one should not try to compare the intensity of the lines, as the result for the test zone is always fainter than the corresponding control zone. Test results should be read within the window recommended by the manufacturer and under good lighting conditions. The conditions for testing at a nightclub or bar are not ideal. Finally, as shown previously, the performance of test strips vary from lot to lot.

## Conclusions

We recommend the most sensitive test strips to be used to minimize false positive results due to lot variations. A false positive result towards a fentanyl drug analogue is not detrimental, as these drugs can also cause toxic effects. Strips that are positive to drugs and compounds that are not fentanyl analogues will not cause harm but may lead to discarding of viable samples.

## Data Availability

The datasets used and/or analyzed during the current study are not available from the corresponding author on reasonable request.
